# Estimates of the mean difference in orthopaedic randomized trials: obligatory yet obscure

**DOI:** 10.1186/s12874-021-01249-2

**Published:** 2021-03-24

**Authors:** Lauri Raittio, Antti Launonen, Ville M. Mattila, Aleksi Reito

**Affiliations:** 1 The Faculty of Medicine and Health Technology, Tampere University, Arvo Ylpön katu 34, 33520 Tampere, Finland; 2 Department of Orthopaedics and Traumatology, Tampere University Hospital, Teiskontie 35, 33520 Tampere, Finland

**Keywords:** Sample size, Uncertainty, Randomized Controlled Trials as Topic, Orthopaedics, Confidence intervals, Patient reported Outcome measures, Statistical inference, Scientific Inference, Power

## Abstract

**Background:**

Randomized controlled trials in orthopaedics are powered to mainly find large effect sizes. A possible discrepancy between the estimated and the real mean difference is a challenge for statistical inference based on p-values. We explored the justifications of the mean difference estimates used in power calculations. The assessment of distribution of observations in the primary outcome and the possibility of ceiling effects were also assessed.

**Methods:**

Systematic review of the randomized controlled trials with power calculations in eight clinical orthopaedic journals published between 2016 and 2019. Trials with one continuous primary outcome and 1:1 allocation were eligible. Rationales and references for the mean difference estimate were recorded from the Methods sections. The possibility of ceiling effect was addressed by the assessment of the weighted mean and standard deviation of the primary outcome and its elaboration in the Discussion section of each RCT where available.

**Results:**

264 trials were included in this study. Of these, 108 (41 %) trials provided some rationale or reference for the mean difference estimate. The most common rationales or references for the estimate of mean difference were minimal clinical important difference (16 %), observational studies on the same subject (8 %) and the ‘clinical relevance’ of the authors (6 %). In a third of the trials, the weighted mean plus 1 standard deviation of the primary outcome reached over the best value in the patient-reported outcome measure scale, indicating the possibility of ceiling effect in the outcome.

**Conclusions:**

The chosen mean difference estimates in power calculations are rarely properly justified in orthopaedic trials. In general, trials with a patient-reported outcome measure as the primary outcome do not assess or report the possibility of the ceiling effect in the primary outcome or elaborate further in the Discussion section.

## Background

A view widely echoed by scholars, funding bodies and readers is that a well-conducted randomized controlled trial (RCT) should have at least 80 % power to detect statistically significant findings if an estimated mean difference (MD) exists between intervention arms [1]. In orthopaedics, the MD estimate between trial arms is often referred to as a minimal clinical important difference (MCID). MCIDs are established for common patient-reported outcome measures (PROMs) in the hope of achieving alignment between patient values and outcome measures by expressing patient-level change in comparison to health status. These estimates are mean estimates of patient-level change in PROM scores that are anchored to an external question of change in health status or of the distribution of these change scores or both [2–4], although expert panels could formulate as well as a foundation for realistic size of MD estimate [5].

However, the variation in MCID estimates for the same PROM has recently become a study question of its own. Further, a large divergence has also been reported between MCID estimates for the same PROM between heterogeneous patient populations [6] and between seemingly homogenous patient populations [7, 8]. In addition, the methodology and differences in the assessment of MCID estimates have not gone unnoticed as a component of divergencfe in MCID estimates [9]. Furthermore, contrasting the absolute scores of MCID estimates to the level of baseline score, direction of change in health status and objective outcome measures yield contradicted results [10–12]. PROM validation and psychometric studies of outcome measures often use a shorter follow-up period (months) for the assessment of the MCID estimate than that commonly used in RCTs (years). As a result, bias between the estimated and the observed mean difference in outcome assessment may occur if there is a mismatch and the MCID estimate can not be generalized to all follow-up time-points.

PROMs are commonly understood to be continuous outcomes with maximum and minimum values that are treated as linear functions of the ability to function in the specified domain. This enables understandable statistical inferences from the mean values of observations. In a statistical sense, all patients reaching the minimum or maximum value are treated as equals regarding the study question when mean values are compared and MDs estimated [13]. However, some PROMs are known to suffer from the ceiling and floor effects because the major proportion of patients end up with the minimum or maximum score in the PROM scale [14, 15]. Moreover, in many circumstances, the longer the follow-up, the closer to the best or worst value of the PROM scale the mean value of the sample may be, which could suppress the possibility of detecting differences between the groups.

In this study, we investigate the rationale behind the choice of the MD estimates (MD_est_) in RCTs published in eight orthopaedic journals between 2016 and 2019. All RCTs using PROM score as the primary outcome, the assessment of the distribution of participant scores, and elaborated on the possibility of ceiling effects and the extent of its hedging in the Discussion section were assessed. In addition, we explore the distribution of the pre-specified or the last follow-up point of the RCTs.

## Methods

### Study selection

We reviewed eight journals focused on clinical orthopaedic research, namely the Journal of Bone and Joint Surgery; Clinical Orthopaedics and Related Research; the Bone and Joint Journal; the American Journal of Sports Medicine; Arthroscopy; the Journal of Arthroplasty; Knee Surgery Sports Traumatology and Arthroscopy; Acta Orthopaedica.

The electronic Tables of Contents from the 2016 to 2019 volumes of each of the eight journals was searched issue by issue in chronologic order to identify published RCTs. All 1:1 allocated RCTs were included in the analysis. Trials with 3 or more arms were excluded.

### Data extraction

**Fig. 1:**
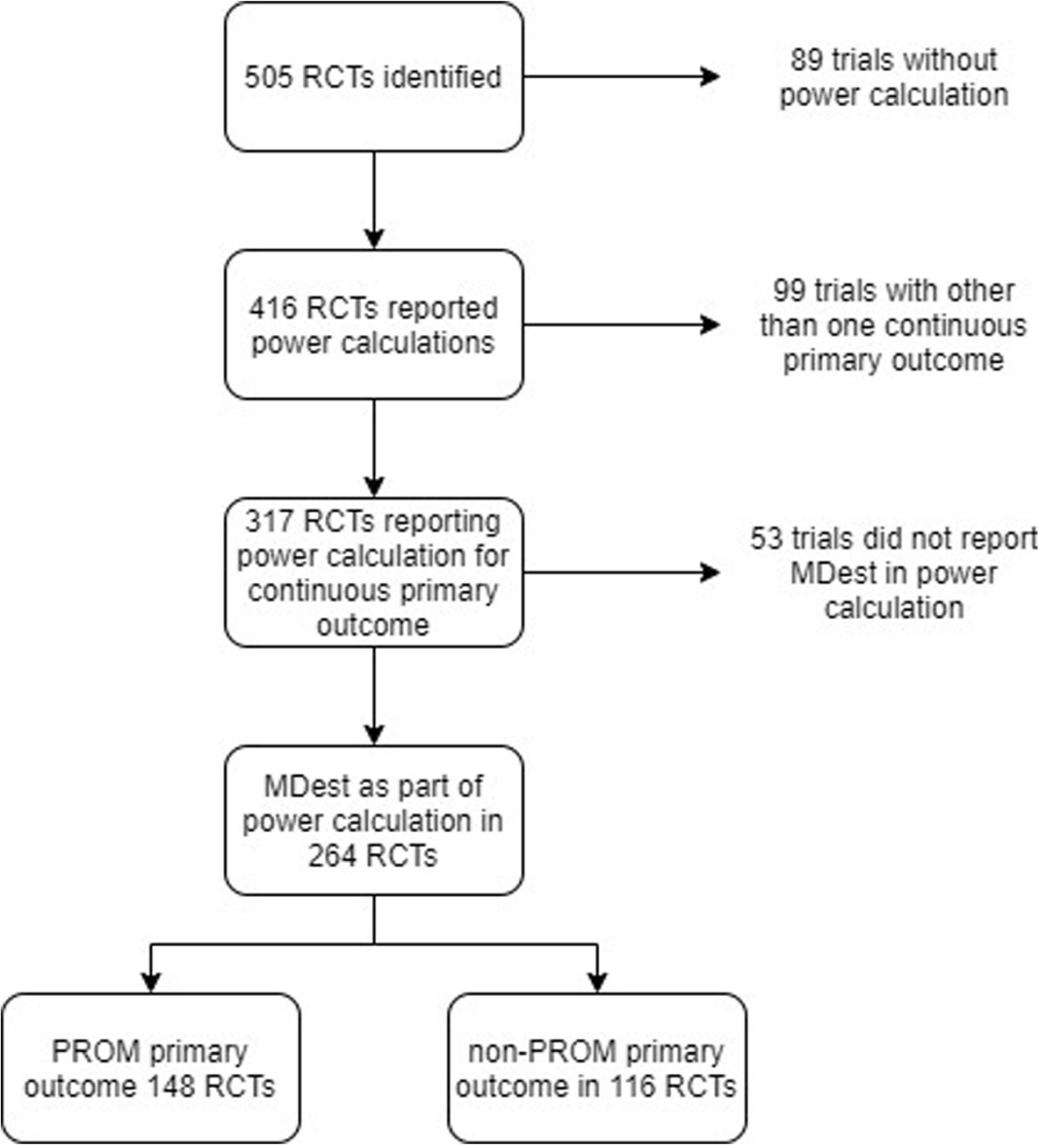
Flow chart of the review and assessment process. Footnotes: *MDest = The mean difference estimate used in power calculation; PROM = Patient-reported outcome measure. **Of the 264 trials that reported the MD_est_ for the power calculation, 76 did not report means and standard deviations for the primary outcome at the pre-specified or the last follow-up time-point

The data on power analysis and observed data estimates from trial data, such as the MD and standard deviation (SD) of the identified trials, were recorded. Figure 1 shows how the sample of eligible results of RCTs was selected. In this study, we regarded the outcome of power calculation as the primary outcome of each RCT. Both the use of power analysis and the type of primary outcome (continuous, binary, several, other) used in the trials were recorded. In addition, we recorded the rationale or reference provided for the chosen MD_est_ if available/extant. The number of patients and the time-point of the pre-specified (or latest) follow-up time-point of the primary outcome data were recorded. For the same time-point, we extracted the means and associated estimate of variability, SD for the primary outcome. If the total SD for the sample or the SD for both trial arms were not reported, the SD_pooled_ was calculated from standard error (SE) or from confidence intervals (CIs), as described in the Cochrane Handbook [16].

The follow-up time-points for the assessment of the results were divided into nine categories merely by convinience: not applicable; 1 day or less, 1 day to 1 week; 1 week to 1 month; 3 months to 1 year; 1 to 3 years; 3 to 10 years and over 10 years. The time-point was categorized as “non applicable” for outcomes such as length of stay in hospital, blood loss in operating room or for concentrations of biological markers in blood samples.

The results section of each RCT was searched for any distribution assessment of the primary outcome scores in addition to mean (SD) or median (intra-quartile range (IQR) or range). These approaches to the distribution of observations in the primary outcome scores were also categorized into nine applicable groups post hoc.

Each outcome score was classified as either patient-reported outcome measure (PROM) or non-patient-reported outcome measure (non-PROM). If the outcome measure only had patient-reported items or a combination of patient-reported and clinician-reported items, the outcome measure was classified as a PROM. For each PROM scale, the best and worst scores were assessed. At the pre-specified or last follow-up time- point, the weighted mean and the SD_pooled_ of the primary outcome of the trial arms were converted to percentage points of the PROM scale to assess the possibility of the ceiling effect. For instance, if the PROM scale was from 0 to 50 with the best score of 50, then the weighted mean of 40 points was converted to be an 80 % of the ceiling effect. Thus, 100 % exhibited the best score (ceiling value) of the PROM scale. Weighted mean +/- 1 SD_pooled_ of the primary outcome, i.e., the weighted mean of 85 % +/- 20 % (20 % = 1 SD of the primary outcome) were reported for eligible trials. Under normal distribution, approximately 16 % of values are greater and smaller than 1 SD of the mean.

The observed effect size of the primary outcome (MD divided by the between group SD) was compared against the estimated effect size in power calculation and cross-tabulated against the follow-up time.

In addition, we explored the Discussion section of each article for hedging statements and elaboration of possible ceiling effect in the primary outcome of the PROM primary measures. All data were extracted from the trials by LR. In addition, AR extracted and thus duplicated the assessment of data of the rationale/reference for the MD_est_ in power calculations and any discrepancies were solved by discussion.

Frequencies were calculated for the assessed outcomes and crosstabulations between the selected outcomes.

## Results

**Fig. 2:**
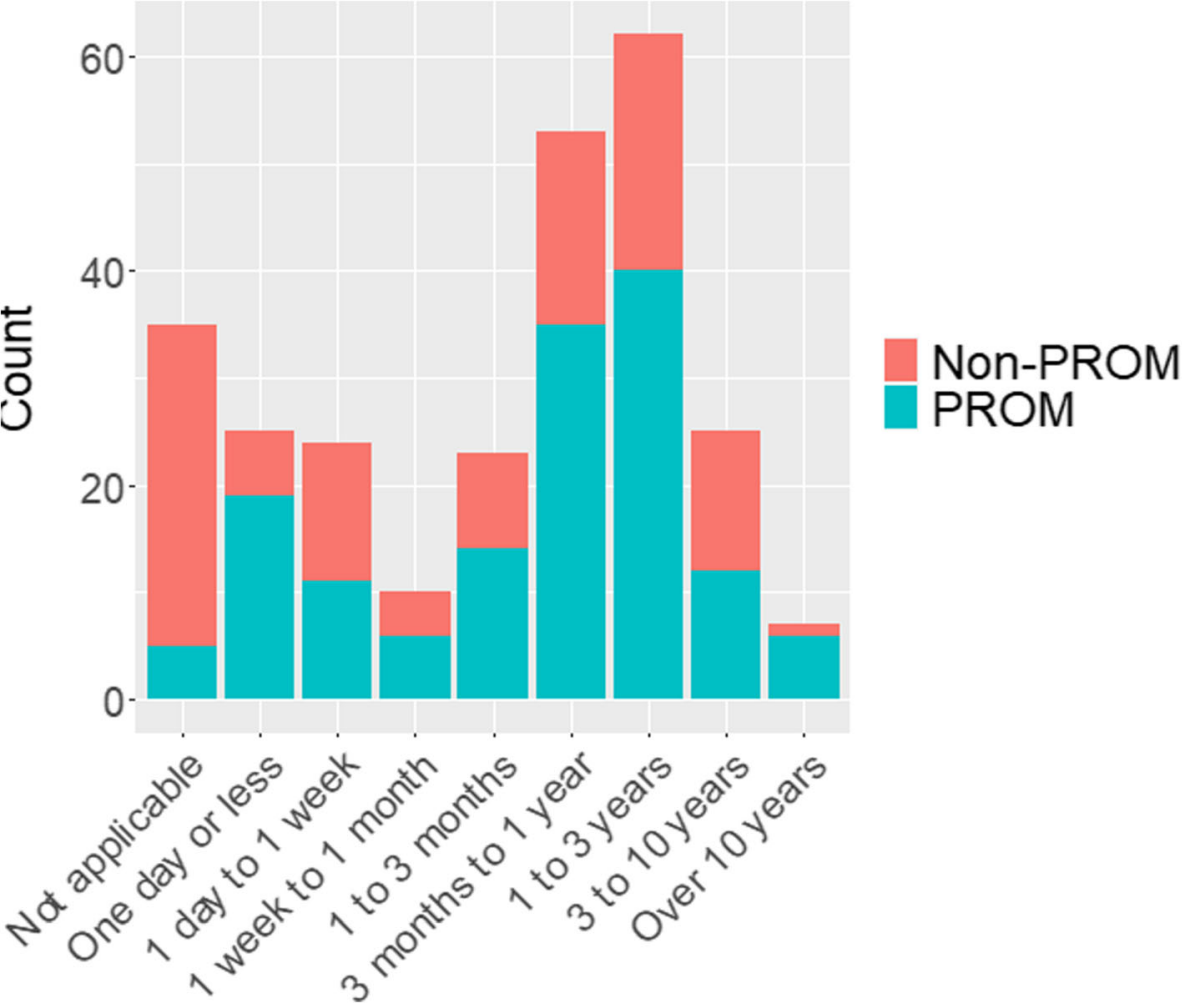
The distribution of the pre-specified or the last follow-up timepoint in 264 RCTs

Of the 505 RCTs identified, 264 RCTs reported the power calculation for one continuous primary outcome, and these were included in the analysis (Fig. 1). Of these 264 RCTs, 148 (56 %) trials used a PROM and 116 (44 %) trials used a non-PROM primary outcome. The pre-specified or the last follow-up time-points were weighted towards one year or more of follow-up and 35 trials had no applicable follow-up point, such as the blood loss, as the primary outcome measured in the operating room (Fig. 2). For 64 % of the RCTs, the follow-up point of the assessed results was measured at 3 months or more. Longer follow-ups were seen in trials with a PROM primary outcome than those with a non-PROM primary outcome (Fig. 2). Of the 50 RCTs with a PROM outcome as the primary outcome and three months or less of follow-up, 41 RCTs assessed pain in the visual analogue scale or in the numerical rating scale as the primary outcome.

The rationale or reference alongside the power calculation for MD_est_ was provided in 108 (41 %) trials (Table 1). RCTs with a PROM (47 %) as the primary outcome reported the rationale or reference for MD_est_ more often than those RCTs with a non-PROM (34 %) primary outcome. Of all trials, the three most common rationales or references for MD_est_ were MCID (43, 16 %), observational studies on the same subject (22, 8 %) and the ‘clinical relevance’ (or ‘clinical judgement’ or ‘clinical experience’) of the authors (15, 6 %) (Table 1). One RCT, using blood loss in arthroplasty surgery, based the MD_est_ on the systematic review of the subject. One trial based the MD_est_ proportional to the MCID estimate of the PROM used in the trial.

**Table 1:** Rationale or reference provided for the mean difference (MD) estimate used in power calculation in 264 RCTs

Rationale or reference provided for the estimate of the mean difference in power calculation	Number of RCTs (%)
No rationale or reference	156 (59 %)
Provided some rationale or reference	108 (41 %)
**Rationale or reference provided by PROM and non-PROM RCTs**	
RCTs with PROM primary outcome	69/148 (47 %)
RCTs with non-PROM primary outcome	39/116 (34 %)
**The rationale or reference provided in power calculation**	
MCID or MDC or PASS from psychometric study for PROM	43 (16 %)
Validation study of the PROM	3 (1 %)
Standardized effect size	2 (1 %)
Systematic review of subject	1 (0.4 %)
RCTs	11 (4 %)
Observational studies	23 (9 %)
Pilot study or pilot data	5 (2 %)
Case series	1 (0.4 %)
‘Clinical relevance’, ‘Clinical judgement’, ‘Clinical experience’	15 (6 %)
Proportional difference to the mean value of historical cohort or MCID (27–40 % decrease)	4 (2 %)

A substantial majority of the RCTs (84 %) assessed the distribution of the primary outcome only by means (SD), medians (IQR) or ranges (Table 2). Of the other distribution assessments of the patient-level observations, categorization of the observations and charts of box-plots were both reported in 19 (7 %) trials. In a few trials, patient-level pre- and post-scores were compared to the value of minimal important change in the PROM or labeled as ‘responders’ of the intervention.

**Table 2:** Presence of distributional assessments of the primary outcome in 264 RCTs

The category of distribution presentation on the primary outcome data	Number^a^ of RCTs (%)
Only mean (SD) or median (IQR) or range provided	209 (79 %)
**Reported one or more of the assessments categorized as below**	58 (16 %)
Categories	19 (7 %)
Box-plots	19 (7 %)
Patient-level change for all patients	2 (1 %)
Number of observations at ceiling minimum or maximum value of the PROM	2 (1 %)
Number of ‘outliers’	8 (3 %)
Number of ‘responders’	3 (1 %)
Number of patients ‘gained’ MIC or MDC	3 (1 %)
Scatter plot of observations	1 (0.4 %)

Footnotes: ^a^RCTs reporting information on multiple categories of distribution presentations were counted for each category provided. *SD* standard deviation; *IQR* intra-quartile range; *PROM* patient-reported outcome measure; *MIC* minimal important change; *MDC* minimal detectable change

**Fig. 3:**
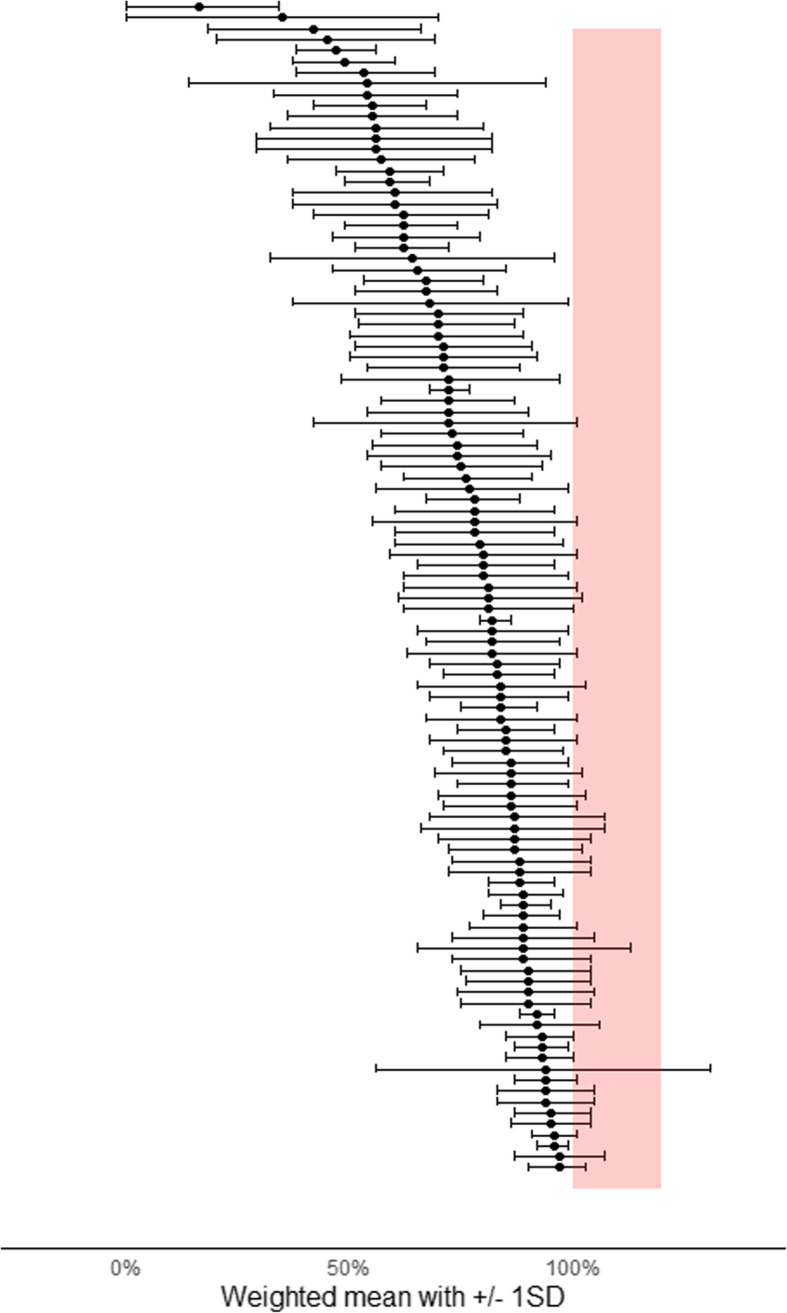
The weighted mean +/- 1 standard deviation of the primary outcome in 107 RCTs with a patient-reported outcome measure

The weighted mean of two trial groups +/- 1 SD of the primary outcome at the pre-specified or the last follow-up time-point (SD_pooled_) were compared against the best value in the PROM scale in 107 RCTs that had eligible mean and SD values (Table 3). In general, the longer the follow-up the closer the weighted mean was to the best score in the PROM scale. In one third of the trials, the weighted mean was over 85 % of the best score in the PROM scale. In 38 (36 %) trials, the weighted mean plus one SD of the primary outcome reached over 100 % in the PROM scale (Fig. 3). In only five of the 148 trials with a PROM primary outcome was a possible ceiling effect discussed in the Discussion section and three of them used the Harris Hip Score.

**Table 3:** The distribution of the weighted means of the trial arms of the primary outcome in percentage points of the best value in the PROM scale categorized by the follow-up time-point in 107 RCTs

Weighted mean in percentages of the best score in the PROM scale	Number of RCTs (%)	Follow-up time not applicable	Follow-up under 3 months	Follow-up between 3 and 6 months	Follow-up between 6 and 12 months	Follow-up over 12 months
<=60 %	19 (18 %)	1 (5 %)	11 (58 %)	3 (5 %)	1 (5 %)	3 (16 %)
Over 60 % and < = 75 %	24 (23 %)	2 (8 %)	6 (25 %)	7 (29 %)	1 (4 %)	8 (33 %)
Over 75 % and < = 85 %	26 (25 %)	2 (8 %)	3 (12 %)	2 (8 %)	9 (35 %)	10 (38 %)
Over 85 % and < 90 %	23 (23 %)	0	4 (17 %)	3 (13 %)	4 (17 %)	12 (52 %)
Over 90 %	15 (14 %)	0	0	2 (13 %)	2 (13 %)	11 (73 %)

The ratio of the observed and the estimated effect size was compared in 113 (43 %) trials and cross-tabulated with the follow-up time (Table 4). Trials with longer follow-up time tended to have lower ratio of observed effect sizes to the estimated effect size. The median of ratios ranged between 0.21 and 0.81, however, the number of trials in each group was low (13–38).

**Table 4:** The ratio of observed and estimated effect size in 113 randomized trials

Ratio of observed effect size and estimated effect size in power calculation	Follow-up time not applicable	Follow-up under 3 months	Follow-up between 3 and 6 months	Follow-up between 6 and 12 months	Follow-up over 12 months	Total number of RCTs (%)
Median (IQR)	0.87 (0.7–1.1)	0.51 (0.2–0.8)	0.39 (0.2–0.6)	0.21 (0.2–0.5)	0.28 (0.1–0.6)	0.41 (0.2–0.8)
< 0.5	1 (8 %)	15 (48 %)	10 (63 %)	12 (80 %)	27 (71 %)	65 (58 %)
0.5-1.0	6 (46 %)	9 (29 %)	3 (19 %)	1 (7 %)	7 (18 %)	26 (23 %)
1.0-1.5	5 (38 %)	4 (13 %)	3 (19 %)	2 (13 %)	2 (5 %)	16 (14 %)
> 1.5	1 (8 %)	3 (10 %)	0	0	2 (5 %)	6 (5 %)

## Discussion

In this study, we investigated the rationales for the MD_est_ used in power calculations, the distribution of follow-up length and the possibility of ceiling effect in RCTs in eight major orthopaedic journals published between 2016 and 2019. The assessment of these factors were divided based on the classification of the primary outcomes in PROM and non-PROM outcomes. Less than half of the RCTs provided rationale or reference for the chosen MD_est_ in power calculation, and the length of follow-up for most studies was over 3 months. Moreover, the distribution of observations in the primary outcome was rarely assessed beside of mean (SD) or median (IQR) values. The possibility of the ceiling effects was evident in a substantial proportion of the trials. However, no elaboration of this was given in the Discussion sections in majority of these trials.

Rationales or references for MD_est_ was provided more often for PROM than non-PROM outcomes (47 % and 34 % of trials), and the most common justification was MCID derived from a psychometric study of the PROM (Table 2). Referencing observational studies, other RCTs and ‘clinical relevance’ without more elaboration of the subject were common justifications for the MD_est_ used in power calculations. Indeed, only one RCT derived the MD_est_ from the effect size found in a systematic review. In rehabilitation trials of low back pain, 48 % of the RCTs referenced the source to calculate the MD estimate and under half of the trials discussed the clinical relevance of treatment effects when results did not reach statistical significance [17]. In the similar vein, the ethics committee documents of RCTs in United Kingdom found that 43 % justified the treatment effect size and 12 % discussed the clinical importance of it [18]. By selecting observational studies with large treatment effects or by considering only the statistical significant results of these studies, one is extremely likely to end up with an inflated treatment effect estimate [19, 20].

Previously, we have shown that orthopaedic RCTs [21] have low power to find small or moderate effect sizes (Cohen’s d = 0.2 or 0.5) and the reporting of sample size calculations often omits some of the essential parameters of the calculation and is incomprehensive [22]. The problems of low power and reliance on inference with p-values has been elaborated by many authors [21, 23, 24]. Moreover, large treatment effects were extremely rare in over 11 000 meta-analyses in the Cochrane database, and the average power of a single RCT was just 9 % to find the associated effect in respective meta-analysis [25]. We are, however, aware of the external requirements that expect RCTs to show an adequate, 80 % or more, power for funding bodies, ethical committees and perhaps even for publishers. Thus, basing the MD_est_ on feasibility rather than on the context of the investigated phenomenon has been called the ‘sample size samba’ [1]. In this light, a sample size derived from the desired precision of the results, the width of the confidence intervals of MD, seems a pleasant alternative [26, 27]. Furthermore, this could enhance to facilitate an effect size estimation approach over the binary ‘yes or no’ framework of the null hypothesis significance testing in the context of the interpretation of trial findings [28].

In our study, the pre-specified or the last follow-up time-point was longer for RCTs with a PROM primary outcome than RCTs with a non-PROM primary outcome. Most of the RCTs with PROM primary outcomes, with 3 months or less of follow-up, were investigating pain scores between trial arms, whereas one third of all the trials with a PROM primary outcome had more than one year of follow-up. However, PROM validation and the psychometric studies of outcome measures often use short-term follow-up for the assessment of the MCID estimate. Moreover, the psychometric properties of PROMs from short-term evaluation cannot be generalized to long-term follow-up without strong assumptions. This is especially concerning for PROMs with known ceiling effects because all observations reaching the ceiling level (the best or worst score in the PROM) are treated as equals in a statistical sense. The true nature of many of the continuous outcomes, such as physical ability to function, cannot have clear ceiling values, even if the PROMs can. Other methods exist, and are outside the scope of this article, that are needed to enhance the validity and reporting of the PROM results are in example [29]; blinded outcome assessment when feasible, focus on what were the comparisons and circumstances in the validation studies of the PROMs and evaluate how the trial population fits to it, reporting outcome assessment methods and reference validation studies appropriately.

The ratio of observed effect sizes to the estimated effect sizes in power calculated was investigated according to the idea that longer follow-up time could decrease the chances to find large differences in MD estimates (Table 4). In general tendency, the longer the follow-up the smaller the ratio between observed and estimated effect sizes. This finding may have many different causes and explanations, but in which the ceiling effect in the primary outcome in trials with longer follow-up time could play some role. More striking is the result that in only 43 % of all the included trials with power calculation for one continuous outcome we were able to compare the observed and the estimated effect sizes. The level of reporting primary outcome data in text and tables is far from optimal.

In a substantial majority (84 %) of the RCTs, no assessment of the distribution of observations other than the means (SD) or medians (IQR and range) of the primary outcome was found in the text, figures or tables. The limits on word counts, figures and tables restrict the assessment of distributions in greater detail, but we argue that the presentation of the information of the observations at the best or at the worst value in the PROM scale is feasible and important. The categorization of the observations and box-plot figures were the most common methods of reporting the distribution of the observations, if extant.

In order to estimate the possibility of ceiling effects in RCTs, we compared the weighted mean of the trial arms in the pre-specified or the last follow-up to the best possible score in the PROM scale. The weighted mean +/- 1 SD of the primary outcome was converted to percentages and 100 % exhibited the ceiling of the PROM scale. One third of the trials had a cross-over between the weighted mean + 1 SD and the ceiling value of the PROM scale. Under normal distribution, approximately 16 % of values are greater and smaller than 1 SD of the mean. In general, the lengthier the follow-up, the closer to the best value in the PROM scale the weighted mean was. We explored the discussion sections of the RCTs and found that only 5 out of 148 trials used a PROM primary outcome to elaborate on the possibility of ceiling effect in the primary outcome.

This investigation of the rationales of the MD_est_ used in power calculations and the handling of possible ceiling effects in orthopaedic RCTs has several limitations. We focused only on eight high impact factor orthopaedic journals. The assessment of the justification of the MD_est_ for power calculations was imperfect as the subject of rationalizing was often addressed by the provision of one or two references to other articles on the same subject. Therefore, there might be other reasons, such as the feasibility of the sample size, rather than the genuine estimates of possible MDs between trial arms. Moreover, it should be acknowledged that many of the very important issues about using PROMs in RCTs can not be reduced to the size of the MD estimate; the availed outcome measures should be valid, sensitive and interpretable in the very specific context of the trial and reported appropriately [30]. By unifying the outcome measures assessed in the common clinical contexts studied could make one leap forward [31, 32].

## Conclusions

In many orthopedic RCTs, the logic behind the choice of MD estimate is missing. Moreover, the possibility of ceiling effect was not evaluated in most of the RCTs. The size and justification of the MD estimate is of the utmost importance in the assessment of the ethicality of the trial. If the size of the estimated MD is a lot greater than the true MD, even a set of p-values will not provide reliable statistical inference. Thus, the assessment of the effect size and its uncertainty in confidence intervals results in more trustworthy inferences from the trial if the true MD estimate falls between the estimated MD and the null. By combining the observed data with the previous literature on the subject, the MD of interest can be estimated more precisely. We praise of quantitative answers for quantitative questions, such as the observed confidence interval of MD over the binary answer of p-value significance testing. This “estimation approach” is easy to aggregate in the design of the sample size and in the interpretations of the trial results.

## Data Availability

The .csv and .xlsx files of the extracted data are provided as supplementary material of the article.

## Abbreviations

CI: Confidence intervals
CI_MD_: Confidence intervals of the mean difference
IQR: Intra-quartile range
MCID: Minimal clinically important difference
MD: Mean difference
MD_est_: Mean difference estimate
PROM: Patient-reported outcome measure
RCT: Randomized controlled trial
SD: Standard deviation
SD_est_: Standard deviation estimate
